# Patterns of Malnutrition in Children Aged Six Months to Five Years: A Community-Based Cross-Sectional Study in Eastern Uttar Pradesh

**DOI:** 10.7759/cureus.89945

**Published:** 2025-08-12

**Authors:** Anil Kumar, Mudit Chauhan, Harish C Tiwari, Pooja Pathak, Navin Kumar, Raja Rao Tamma

**Affiliations:** 1 Community Medicine, Uma Nath Singh Autonomous State Medical College, Jaunpur, IND; 2 Community and Family Medicine, Baba Raghav Das (BRD) Medical College, Gorakhpur, IND

**Keywords:** children aged six months to five years, malnutrition, stunting, underweight, wasting

## Abstract

Background: Children are the most important asset of the country. They are the future of their country. Malnutrition commonly affects all groups in a community, but the most vulnerable are infants and young children because of their high nutritional requirements for growth and development. The present study was planned to estimate the prevalence of malnutrition in this area and the factors contributing to it in this geographical region. It may be helpful in improving the nutritional status of children in this area. The present study was undertaken to assess the prevalence and determinants of underweight among children aged six months to five years in Eastern Uttar Pradesh.

Methods: A study was conducted in Eastern Uttar Pradesh. Among the children aged six months to five years, the targeted study population of 370 participants was selected from the four villages of Block Primary Health Center (PHC) after implementing the exclusion criteria. The study design was cross-sectional, and a multistage random sampling technique was used. Data were collected through a house-to-house survey by interviewing the mother/father/guardian of the children. The nutritional status of preschool children was evaluated using the 2006 World Health Organization (WHO) Growth Standards. Z-scores were computed for weight-for-age (underweight), height-for-age (stunting), and weight-for-height (wasting). To compute the Z-scores, the individual value was subtracted from the median reference value and divided by the standard deviation of the reference population. A Z-score below -2 SD from the median was used to identify malnutrition, with Z-scores below -3 SD indicating severe undernutrition.

Results: The present study was conducted with 370 children aged six months to five years. Of the 370 participants, 94.6% were Hindu, while 5.4% were Muslim. Among the participants, 48.4% were male and 51.6% were female. The majority of fathers had an education up to middle school, while most mothers were educated up to primary school level (35.1%), and 27.6% were illiterate. A significant portion of fathers were semi-skilled workers, and most mothers were unemployed. The majority of participants belonged to lower-middle socioeconomic status and lived in nuclear families. The prevalence of underweight, stunting, and wasting among the children was 30.8%, 30.5%, and 16.5%, respectively.

Conclusions: Though many mothers were illiterate or educated up to primary school, malnutrition was more prevalent among their children. Mothers should be provided with special counseling regarding breastfeeding and complementary feeding.

## Introduction

The development and productivity of any nation cannot be achieved if its children are not properly nourished. Proper nutrition is a crucial first step in enhancing quality of life. Nutrition plays a vital role in the physical, mental, and emotional development of children. Significant attention has been given to ensuring proper nutrition for growing populations, particularly during the formative years of life [1]. In India, the number of underweight children is nearly twice that of sub-Saharan Africa [2]. Malnutrition impacts all groups within a community, but infants and young children are particularly vulnerable due to their increased nutritional needs for growth and development [3]. The analysis of National Family Health Survey-4 (NFHS-4) data revealed a strong correlation between under-five child mortality rates and child underweight rates. According to the NFHS-4, the prevalence of underweight, stunting, and wasting was 35.7%, 38.4%, and 21%, respectively, compared to 42.5%, 48%, and 19.8% in the National Family Health Survey-3 (NFHS-3). This indicates a reduction in the prevalence of malnutrition in India [4,5]. According to the recent National Family Health Survey-5 (NFHS-5) (2019-2021) report, nutrition indicators for children under five years have shown improvement compared to NFHS-4 (2015-2016). Stunting has decreased from 38.4% to 35.5%, wasting has reduced from 21% to 19.3%, and the prevalence of underweight has dropped from 35.8% to 32.1% [6]. Gorakhpur district is one of the important districts of Eastern Uttar Pradesh, which has poor health indicators as per NFHS-4. Health and other services are improving day by day in this area, and NFHS-5 data show some improvement in the nutritional status of children in this area. The present study aims to estimate the prevalence of malnutrition among children between six months and five years.

## Materials and methods

This community-based cross-sectional study was conducted in the Chargawan block of Gorakhpur, Uttar Pradesh, India, over a one-year period from August 1, 2021, to July 31, 2022. The study population comprised children aged between six months and five years residing in the Chargawan block, and the individual unit of study was each child within this age group. A multistage random sampling technique was employed to select the participants for the study, ensuring representativeness across different areas within the block.

Inclusion criteria

Included in the study were children aged between six months and five years at the time of the study and children who reside in rural areas of Eastern Uttar Pradesh, specifically within the selected study locations. Parents or guardians of the children must provide informed consent to participate in the study. Children with any nutritional status (underweight, stunting, wasting, and normal) can be included to assess the full range of malnutrition patterns.

Exclusion criteria

Excluded from the study were children outside the age range of six months to five years (e.g., infants younger than six months or children older than five years), children who do not reside in the rural areas of Eastern Uttar Pradesh, particularly those from urban areas or non-local regions, children with any acute severe illness at the time of the study (e.g., fever, infections, severe dehydration) that could interfere with nutritional assessment, children whose parents or guardians did not provide informed consent for participation in the study, children diagnosed with severe chronic medical conditions (e.g., congenital metabolic disorders) that are known to have significant changes in nutritional patterns and are outside the scope of this study, and children undergoing specialized nutrition interventions (e.g., receiving intensive medical treatment for malnutrition) outside of general community healthcare.

Sample size

The prevalence of undernutrition in children below five years was 35.7% [4]. The sample size was calculated using the formula N=Z²pq/l², where Z=1.96 (for 95% confidence level), p=35.7 (prevalence in percentage), q=100-p=64.3, and l=5 (allowable error in percentage). A minimum sample size of 353 is obtained; after rounding off, 370 samples were included in the study.

Study tools

A pre-tested and pre-designed questionnaire was designed at the Department of Community Medicine of Baba Raghav Das (BRD) Medical College to assess the nutritional status of children aged between six months and five years in the field practice area of BRD Medical College, Gorakhpur. Height was measured using a stadiometer for children aged two years and above, and an infantometer was used for children under two years of age. Weight was measured using a calibrated digital weighing scale for children aged two years and above, and a Salter's hanging spring scale was used for infants and younger children who could not stand unaided.

Methodology

The help of a health worker (ASHA) and local people was taken to find the address of selected children. Before the collection of data from the study subject, written consent was taken from their parents/guardians in Hindi after explaining the procedure and purpose of the study. Complete confidentiality and anonymity of the respondents were maintained. Each participant of the sample population was directly interviewed with the help of a pre-tested and pre-designed questionnaire. A total of 370 participants were interviewed.

Measurement of weight and height

Children's weight was measured using a calibrated digital scale for those over two years and a Salter spring balance for those under two. They were weighed without footwear and minimal clothing, with body weight evenly distributed on the scale. Weights were recorded to the nearest 0.5 kg, and zero error was adjusted before each measurement [7].

Height was measured using a stadiometer with children standing barefoot, in minimal clothing, and aligned in the Frankfurt plane. The measurement was recorded to the nearest 0.5 cm. For children under two years, supine length was measured using an infantometer. The child's head was positioned against the fixed headboard, knees extended, and feet at right angles to the lower legs. The footpiece was adjusted to contact the heels, and the length was measured to the nearest 0.5 cm [7].

Assessment of nutritional status using the anthropometry method

The nutritional status of preschool children was evaluated using the 2006 World Health Organization (WHO) Growth Standards. Z-scores were computed for weight-for-age (underweight), height-for-age (stunting), and weight-for-height (wasting). To compute the Z-scores, the individual value was subtracted from the median reference value and divided by the standard deviation of the reference population. A Z-score below -2 SD from the median was used to identify malnutrition, with Z-scores below -3 SD indicating severe undernutrition [8,9].

Statistical analysis

Data obtained after the interviews of the participants were entered into a Microsoft Excel sheet (Microsoft Corporation, Redmond, Washington, United States). The master chart was prepared. Data was analyzed, and an appropriate statistical test was applied by using IBM SPSS Statistics for Windows, Version 26.0 (Released 2019; IBM Corp., Armonk, New York, United States). All the necessary help, advice, and counseling were given to the parents of the study subjects aged six months to five years who were found to be malnourished.

Ethical clearance

Ethical permission was obtained from the College Research Council of BRD Medical College (approval number: 58/CRC/2021), and written informed consent was obtained from the parents or guardians of each child.

## Results

The distribution of children aged six months to five years (N=370) based on their socio-demographic profile and personal history is as follows. Among the children, 114 (30.8%) were in the age group of 48-59 months, followed by 84 (22.7%) in both the 12-23- and 24-35-month groups. The gender distribution was nearly even, with 179 (48.4%) male and 191 (51.6%) female children. A majority of the children (350, 94.6%) belonged to the Hindu religion, while 20 (5.4%) were Muslim. In terms of caste, 258 (69.7%) were from the Other Backward Classes (OBC), 72 (19.5%) from Scheduled Castes/Scheduled Tribes (SC/ST), and 40 (10.8%) from the General category. The socioeconomic classification of the families revealed that 204 (55.1%) of the children belonged to lower-middle-class families and 119 (32.2%) came from middle-class families. The educational level of the parents varied, with 120 (32.4%) fathers and 130 (35.1%) mothers having only completed primary school. A significant proportion of fathers (243, 65.7%) were semi-skilled workers, while the majority of mothers (359, 97%) were unemployed. Regarding family structure, 263 (71.4%) of the children lived in families with fewer than five members, and 249 (67.3%) of them belonged to nuclear families. The birth order indicated that 245 (66.2%) of the children were born in families with two or fewer children. Additionally, the majority of children (269, 72.7%) had a birth weight of 2.5 kg or more, while 101 (27.3%) were born with a lower birth weight of less than 2.5 kg (Table 1).

**Table 1:** Distribution of children six months to five years of age according to their socio-demographic profile and personal history (N=370) Gen: general; OBC: Other Backward Classes; SC/ST: Scheduled Castes/Scheduled Tribes

S. no.	Variables		Frequency (n)	Percentage (%)
1	Age (in months)	6-11	23	6.2
		12-23	84	22.7
		24-35	84	22.7
		36-47	65	17.6
		48-59	114	30.8
2	Gender	Male	179	48.4
		Female	191	51.6
3	Religion	Hindu	350	94.6
		Muslim	20	5.4
4	Caste	Gen	40	10.8
		OBC	258	69.7
		SC/ST	72	19.5
5	Socioeconomic classification	Upper	13	3.5
		Upper middle	17	4.6
		Middle	119	32.2
		Lower middle	204	55.1
		Lower	17	4.6
6	Education of the father	Graduate and above	17	4.6
		Intermediate	13	3.5
		High school	20	5.4
		Middle school	105	28.4
		Primary school	120	32.4
		Illiterate	95	25.7
7	Education of the mother	Graduate and above	27	7.3
		Intermediate	26	7
		High school	67	18.1
		Middle school	18	4.9
		Primary school	130	35.1
		Illiterate	102	27.6
8	Occupation of the father	Clerical, shop owner, farmer	11	3
		Skilled worker	23	6.2
		Semi-skilled worker	243	65.7
		Unskilled worker	75	20.3
		Unemployed	18	4.9
9	Occupation of the mother	Skilled worker	3	0.8
		Semi-skilled worker	3	0.8
		Unskilled worker	5	1.4
		Unemployed	359	97
10	No. of family members	<5	263	71.4
		≥5	107	28.6
11	Type of family	Nuclear family	249	67.3
		Joint family	121	32.7
12	Birth order	≤2	245	66.2
		>2	125	33.8
13	Birth weight (kg)	≥2.5	269	72.7
		<2.5	101	27.3

Among 370 children, 256 (69.2%) were classified as having a normal weight for height, and 114 (30.8%) were underweight. However, 91 (24.6%) of the children were found to be moderately underweight, while 23 (6.2%) were classified as severely underweight. This indicates that while the majority of children had a normal weight, a notable proportion experienced varying degrees of underweight, highlighting potential concerns regarding nutrition and growth among the study population. The prevalence of stunting among the study subjects shows that 257 (69.5%) were classified as having normal height for their age, and 113 (30.5%) were stunted, while 84 (22.7%) were moderately stunted, and 29 (7.8%) were severely stunted. This indicates that while most children had normal height, a significant proportion were affected by stunting, with a smaller group experiencing severe stunting, reflecting potential concerns regarding long-term growth and nutritional status in the study population.

**Figure 1:**
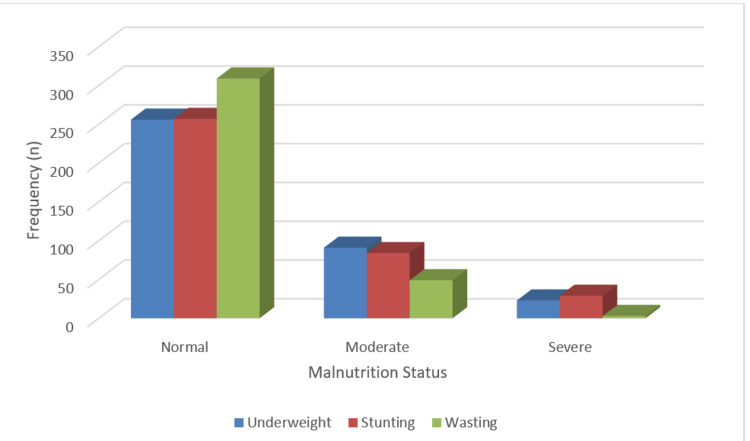
Distribution of study subjects according to type of malnutrition status (N=370)

Among the 370 children, 309 (83.5%) were classified as having normal weight-for-height, indicating no signs of wasting, while 61 (16.5%) were identified as wasted. However, 49 (13.2%) were moderately wasted, and 12 (3.2%) were severely wasted. This highlights that while the majority of children had a normal nutritional status, a smaller yet significant proportion experienced varying degrees of wasting, which raises concerns about their immediate nutritional health (Figure 1).

## Discussion

This study highlights a significant burden of malnutrition among children aged six months to five years in the Chargawan block of Gorakhpur, Uttar Pradesh, India. Among the 370 children studied, 114 (30.8%) were underweight, 113 (30.5%) stunted, and 61 (16.5%) wasted, indicating ongoing concerns with both chronic and acute malnutrition.

The underweight prevalence of 114 (30.8%) aligns with findings by Navya and Udayakiran [3] and Chawla et al. [10] and is slightly lower than the NFHS-4 (2015-2016) [4] report for Uttar Pradesh (35.7%). Similarly, the stunting rate observed in our study was 113 (30.5%), which is lower than the national average of 38.4% and the findings reported by Chawla et al. [10], yet it remains considerably high, indicating persistent long-term nutritional deficits. The wasting prevalence of 61 (16.5%) is comparable to findings by Gupta et al. [11] in rural Haryana, Murarkar et al. [12] in Maharashtra, Chittapur et al. [13], and Sarswat et al. [14], indicating acute nutritional stress in this population.

A significant proportion (101, 27.3%) of children were born with low birth weight, a known contributor to undernutrition, as supported by Deshmukh et al. [7]. Additionally, the majority of mothers (359, 97%) were unemployed, and most families belonged to lower or lower-middle socioeconomic classes, factors strongly associated with poor nutritional outcomes [15].

These findings emphasize the urgent need for strengthened nutrition-focused interventions, including maternal education, improved infant feeding practices, and better coverage of growth monitoring through frontline services like Anganwadi centers. Despite ongoing efforts, malnutrition continues to impact child health and development in rural areas and demands sustained, community-level public health action.

## Conclusions

The present study reveals a considerable prevalence of malnutrition among children aged six months to five years in the Chargawan block of Gorakhpur, with 30.8% underweight, 30.5% stunted, and 16.5% wasted. These findings underscore both chronic and acute nutritional challenges faced by children in this rural area. Socioeconomic factors such as low parental education, high maternal unemployment, and low birth weight were significant contributors. Despite existing nutrition programs, the burden of malnutrition remains substantial. Strengthening maternal awareness, improving access to quality nutrition services, and targeted community-based interventions are essential to address and reduce child malnutrition in such settings.
